# Detection and Imaging of Debonding in Adhesive Joints of Concrete Beams Strengthened with Steel Plates Using Guided Waves and Weighted Root Mean Square

**DOI:** 10.3390/ma13092167

**Published:** 2020-05-08

**Authors:** Erwin Wojtczak, Magdalena Rucka, Magdalena Knak

**Affiliations:** Department of Mechanics of Materials and Structures, Faculty of Civil and Environmental Engineering, Gdansk University of Technology, Narutowicza 11/12, 80-233 Gdansk, Poland; magdalena.rucka@pg.edu.pl (M.R.); s168197@student.pg.edu.pl (M.K.)

**Keywords:** adhesive joint, concrete beam, guided waves, debonding, damage detection, damage imaging, root mean square

## Abstract

Strengthening of engineering structures is an important issue, especially for elements subjected to variable loads. In the case of concrete beams or slabs, one of the most popular approaches assumes mounting an external reinforcement in the form of steel or composite elements by structural adhesives. A significant disadvantage of adhesive joints is the lack of access to the adhesive film for visual condition assessment, thus, there is a need for non-destructive diagnostics of these kinds of connections. The aim of this paper was the identification and visualization of defects in adhesive joints between concrete beams and steel plates using the guided wave propagation technique. The initial theoretical and numerical analyses were performed. The experimental wave field was excited and measured by the scanning laser Doppler vibrometry. The collected signals were processed by the weighted root mean square (WRMS) calculation. As a result, 2-D damage maps were obtained. The numerical simulations were performed to corroborate the experimental results. The results showed that the guided waves could be successfully applied in non-destructive diagnostics of adhesive joints between concrete and steel elements. However, the quality of damage visualizations strongly depended on the location of excitation.

## 1. Introduction

Engineering structures are subjected to various loads, e.g., dead loads, live loads, environmental loads, and other, over the entire service life. In many situations, it may be necessary to increase the load capacity. The main reasons are planned changes in the way a structure is used and carrying out rehabilitation and retrofitting works due to degradation of a structure or its natural aging. To achieve higher load capacity, strengthening is commonly used. Various reinforcement systems such as steel elements (plates, rods, and flat bars) or composite elements (carbon tapes, carbon mats, and other fiber reinforcement polymers) are increasingly used [1,2,3]. Such externally bonded reinforcement (EBR) is very often connected to a strengthened structure by adhesive bonding [4]. It is worth noticing, that the technical condition of the connection between joined elements has a great influence on the behavior of the whole structure and, if not properly evaluated, may cause an unexpected collapse.

The existing literature presents many examples of the use of adhesives in connections between concrete and steel [4,5,6,7,8] as well as between concrete and fiber reinforced polymeric (FRP) composites [9,10,11,12,13,14,15,16,17]. Czaderski and Meier [4] proved that the condition of concrete elements strengthened using the EBR technique can be good even after several dozen years of exploitation. However, the monitoring of such structures is crucial because of the possibility of debonding between the substrate and overlay, which is the most common damage type in this type of connections. It should be also noted that debonding is a dangerous defect due to the lack of its visibility, thus the problem of detection of such damage is not a trivial task. The strength of elements reinforced with EBR technique is usually considered as the bond strength between joined elements [15], thus the condition of the adhesive layer is of great importance. Debonding in externally reinforced concrete elements, which is initiated by crack grows, is the aim of the work of many researchers. The reinforcement in the form of steel plates was considered as the protection of concrete beams against failure induced by flexural [6] or shear cracking [5,7,8]. Typical peel-off tests were conducted for concrete beams strengthened with glass fiber reinforced polyester to determine their strength [9]. The influence of carbon fiber reinforced polymers (CFRP) [10], steel reinforced polymers (SRP), and steel reinforced grouts (SRG) [12] on the failure induced by flexural cracking in concrete beams during bending load was also considered. Pre-stressing of CFRP laminates was shown to be the effective method of reduction of debonding in strengthened concrete beams under flexural load [13]. A number of works were conducted based on the shear tests for concrete beams reinforced with SRG [16] and CFRP [17]. The influence of additional CFRP anchorages on the shear strength was also analyzed [14]. The combined influence of normal and shear stresses was investigated for U-shaped CFRP reinforcement covering the bottom and the sides of the beams [11,15].

With the increasing use of adhesive connections to reinforced structures, there is a need to develop efficient techniques of non-destructive detection and imaging of debonding between a substrate and overlay, as for example active thermography [18,19,20,21,22]. A group of the most effective techniques to evaluate the interfacial adhesion consists of the methods utilizing elastic wave propagation. Existing papers have presented numerous successful applications of wave propagation phenomenon for damage identification in civil engineering structures [23,24,25,26,27]. The impact-echo (IE) method, based on the use of low-frequency vibrations, supported with the frequency spectrum analysis and wavelet analysis, was efficiently utilized for the assessment of adhesion in concrete structures [28,29]. Another promising approach, applicable also for the evaluation of adhesive joints, is based on the analysis of wave energy distribution supported with weighted root mean square (WRMS) calculation, e.g., in aluminum [30] and steel connections [31,32]. Recently, ultrasonic waves have also been increasingly used to inspect and evaluate adhesive bonding between concrete elements and externally bonded reinforcement. Castaings et al. [33] analyzed the propagation of a pseudo-Rayleigh wave mode to evaluate the adhesion level between concrete block and glass-epoxy composite plate joined by the epoxy resin, based on the numerical dispersion calculations and experimental measurements. Shen et al. [34] investigated a concrete block strengthened with steel plate joined by epoxy adhesive. Dispersion properties for a three-layer medium were determined by analytical, experimental, and numerical approaches, giving comparable results. Propagation of shear waves was used by Zeng et al. [35] to detect bond slip at the steel–concrete interface in concrete encased I-shape beam under tensile test. Song and Popovics [36] studied the influence of different bonding conditions on dispersion characteristics of fundamental A_0_ and S_0_ wave modes propagating in the steel-clad concrete beams. They observed that attenuation characteristics of guided waves are sensitive to adhesion level at the steel–concrete interface. Li et al. [37] applied the time-reversal method and continuous wavelet transform to evaluate debonding in the CFRP-reinforced concrete blocks. The analysis of strain distribution of wave propagation in numerical models was used as an attempt to imaging of damaged areas; however the exact size and position of defects were not determined. Zima and Rucka [38] presented experimental and numerical results of condition assessment of adhesive joint between concrete beam and steel plate with various states of degradation. The correlation between the amplitude of peak values in wave signals and bonding quality was observed. The numerical guided wave field represented by acceleration magnitudes was used to visualize debonding areas. Mechanical degradation of adhesive connection in concrete–steel specimens during push-out tests was monitored ultrasonically by Rucka [39]. Changes in the interface slip between adherends were treated as an indicator of approaching failure. Chen et al. [40] proposed detecting interfacial debonding in concrete-filled steel tubes using the transient multichannel analysis of surface waves. The differences in dispersion characteristics allowed determining the presence of defects. Liu et al. [41] monitored debonding between CFRP sheet and concrete beam during three-point bending using the pitch-catch wave-based method. The decrease of signal amplitude was observed in the case of damaged connection compared with an intact one. Experimental strain distribution at different loading levels was used to illustrate the development of damaged areas. Interfacial debonding in steel–concrete and steel-epoxy-concrete plates was quantitatively evaluated by Ke et al. [42]. Comparison of signal spectrograms obtained by the short time Fourier transformation of signals obtained allowed determining the debonding range between joined parts. Yan et al. [43] investigated steel–concrete–steel sandwich beams under bending test. Acoustic emission and electromechanical impedance methods were used to evaluate the cracking process and the structural state of analyzed specimens. Giri et al. [44] analyzed the prediction of a gap in concrete–steel and concrete–aluminum connections using partial least squared regression technique based on the Lamb wave propagation phenomenon. Nonlinear Rayleigh waves were used by Ng et al. [45] for debonding detection in CFRP-retrofitted reinforced concrete blocks. Short time Fourier transformation of wave signals allowed preparing location images of defects with different size. Wang et al. [46] detected the severity and type of damage in concrete columns with CFRP sheeting using Fourier transform and wavelet packet energy analysis. Damage and health indices were used to evaluate concrete cracking and debonding initiation. Huo et al. [47] made a review on monitoring of bond-slip in steel–concrete structures presenting well-known wave-based approaches, including active and passive sensing methods. The reported works present an extensive analysis of debonding detection in steel–concrete composite structures. However, the issue of damage imaging was not deeply considered, except some above-mentioned attempts based on the analysis of the guided wave field represented by the wave acceleration or stress distribution. It should be noted, that the determination of actual location, size, and shape of damaged areas has not been performed, notwithstanding these features have a great impact on the actual strength of adhesive connections.

This paper deals with the condition assessment of concrete beams strengthened with steel plates. The novelty of the study is connected with comprehensive theoretical-numerical-experimental analysis of wave propagation in steel–concrete adhesively bonded specimens and the possibility of debonding imaging using WRMS-based ultrasonic diagnostics. The conducted research was divided into two stages. The initial analysis focused on the characterization of the guided wave propagation in a single-layer and a three-layer media. The main part of the study was directed at the damage identification and imaging in adhesive joints of composite beams with different levels of debonding. The guided waves were sensed and recorded in a number of points on the surface of the steel plate using both contact and non-contact measurement techniques. The signal processing technique based on the WRMS calculation allowed detecting and imaging of the damaged areas. The obtained results indicated that the quality of damage maps strongly depended on the location of excitation.

## 2. Materials and Methods

### 2.1. Object of Research

**Figure 1:**
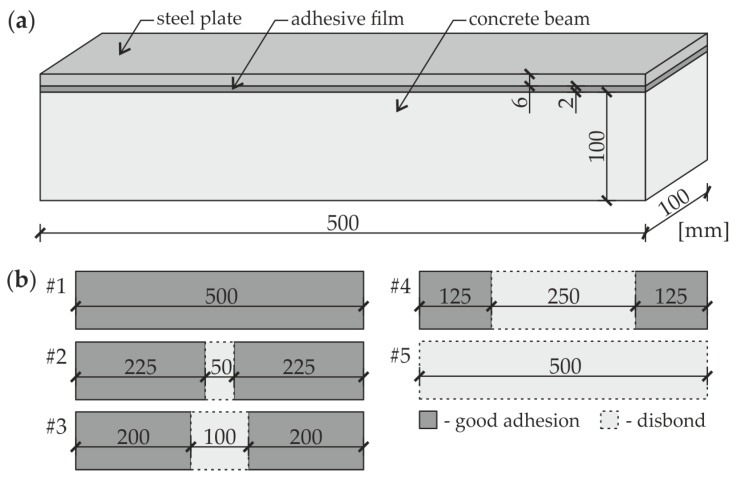
Analyzed specimens: (**a**) geometry of beams and (**b**) variants of defects.

**Figure 2:**
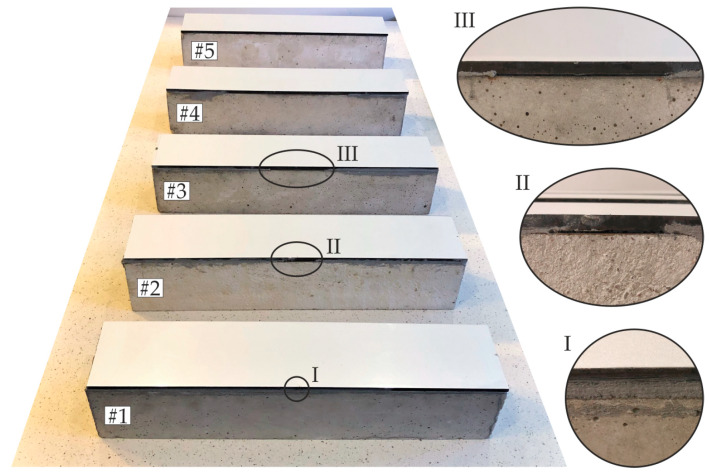
Photograph of prepared specimens (#1–#5) with close-up views of adhesive films in beams #1, #2, and #3.

The object of investigations (Figure 1a) was a beam with a square cross section of 100 mm × 100 mm and a length of 500 mm. The beam was made of concrete class C30/37 and strengthened with a steel plate of dimensions 100 mm × 6 mm × 500 mm fixed to the upper surface of the beam by an adhesive film. Five specimens were analyzed (Figure 1b). The first specimen was an intact composite beam (#1) with a full connection between both elements. Three partially damaged beams (#2, #3, and #4) had defects in the form of a debonding, covering a different area of the joint (relative defect surface was 10%, 20%, and 50%, respectively). The last sample was a steel plate (#5), which was laid freely on the beam to simulate a fully debonded joint. The defects were obtained by sticking a Teflon (PTFE) tape onto the appropriate area of the steel plate. To prevent any unintentional defects, the treatment of each bonded elements was performed. The surface of the concrete beam was dedusted and degreased with Loctite-7063 cleaner (Henkel, Düsseldorf, Germany). The steel plate was treated with a fine abrasive paper (grit size 120) and then degreased. The connection between concrete and steel was provided by the two-component epoxy-based structural adhesive Sikadur 30 Normal (Sika, Baar, Switzerland). The specimens were conditioned in room temperature for seven days after gluing. The photograph of prepared specimens with close-ups for selected defects is presented in Figure 2. The thickness of the adhesive film for specimens (#1–#4) was equal to about 2 mm. It was determined after conditioning as the difference between the overall specimen thickness and the sum of beam and plate thicknesses.

### 2.2. Experimental Procedure

**Figure 3:**
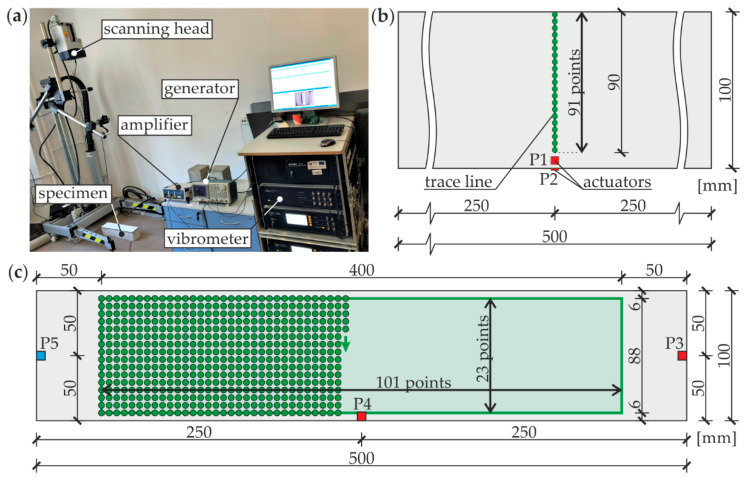
Experimental investigations: (**a**) setup for scanning laser Doppler vibrometry (SLDV) measurements; (**b**) scheme of measurements for dispersion curves determination; and (**c**) scheme for damage identification and visualization measurements.

The experimental measurements of guided waves were carried out by the scanning laser Doppler vibrometry (SLDV) method using the set-up presented in Figure 3a. The input wave signal was generated by the arbitrary function generator AFG 3022C (Tektronix, Inc., Beaverton, OR, USA) and magnified with the use of the high-voltage amplifier PPA 2000 (EC Electronics, Krakow, Poland). The excitation of the guided wave field was provided by the plate piezoelectric actuators NAC2024 and NAC2025 (Noliac, Kvistgaard, Denmark) bonded to the surface of each examined specimen by the petro wax 080A109 (PCB Piezotronics, Inc., Depew, NY, USA). The signals of propagating waves (out-of-plane velocity components) were collected by the non-contact method using the scanning head of the laser vibrometer PSV-3D-400-M (Polytec GmbH, Berlin, Germany) equipped with the VD-07 velocity decoder. The sampling frequency for each measurement was set to 2.56 MHz. The area of scanning was covered with a retro-reflective sheeting to improve the light backscatter.

The samples #1 and #5 were firstly tested to determine dispersion curves in a single-layer and three-layer medium, according to the scheme presented in Figure 3b. For this purpose, the propagation of antisymmetric and symmetric modes was induced by actuators P1 and P2, respectively, both attached to the steel plate. The input signal had the form of a wave packet obtained by the Hanning window modulation of the single-cycle sine function. To obtain a wide frequency spectrum for both excitation types, measurements were conducted five times with the different carrier frequency, equal to 50, 100, 150, 200, and 250 kHz, consecutively. The time-domain wave signals were acquired in 91 points spaced evenly over a straight line with a length of 90 mm, resulting with a resolution equal to 1 mm. The influence of potential reflections of propagating waves at the boundaries, induced by the short path length in relation with beam thickness, was further eliminated by analyzing only the initial part of the collected signals. The main investigations aimed at damage imaging in all beams #1–#5 were conducted with respect to the scheme shown in Figure 3c. Each specimen was tested twice, changing the location of the excitation point (P3 and P4). Excitation signal was a five-peak wave packet obtained from a sinusoidal burst with a central frequency equal to 100 kHz. The signals of propagating waves were collected in 2323 points distributed on the top surface of the steel plate, covering the area of 440 × 88 mm^2^. The scanning was conducted point by point in the regular mesh of 23 rows and 101 columns with a resolution of 4 mm in both directions.

Additional measurements with a contact method were performed to identify the damaged specimens. The input signal was identical to the one used for damage imaging with SLDV (five-cycle sine wave packet with a central frequency of 100 kHz). The piezoelectric actuators were attached at the ends of each beam to excite and acquire wave signals in points P3 and P5, respectively (Figure 3c). The excitation signal was created by the arbitrary waveform generator AFG 3022C (Tektronix, Inc., Beaverton, OR, USA) and then amplified by the high-voltage amplifier A400DI (FLC Electronics AB, Partille, Sweden). The collection of signals was provided by the digital oscilloscope PicoScope 4824 (Pico Technology, St Neots, Great Britain) with a sampling frequency of 20 MHz.

### 2.3. Numerical Modeling

The numerical simulations of the guided wave propagation in the considered specimens were conducted in Abaqus/Explicit software (ver 6.14, Dassault Systemes, Vélizy-Villacoublay, France) applying the finite element method. All structural elements (steel plate, concrete beam and adhesive film) were assumed to be independent parts bonded rigidly by surface to surface tie connection, ensuring compatibility of translational degrees of freedom at contacting nodes. The materials of modelled parts were supposed to meet the requirements of a homogeneous, isotropic and linearly elastic material model. The mechanical properties of each material are presented in Table 1. The transient dynamic analysis was conducted with the use of the central difference method. Total time of calculations was assumed as 2 ms for each simulation. The propagation of wave was analyzed by considering the behavior of models under the concentrated load with varying amplitude, applied in a specific node of a discretized structure. The results of the analyses were out-of-plane (vertical) velocity signals collected in a certain number of nodes.

**Figure 4:**
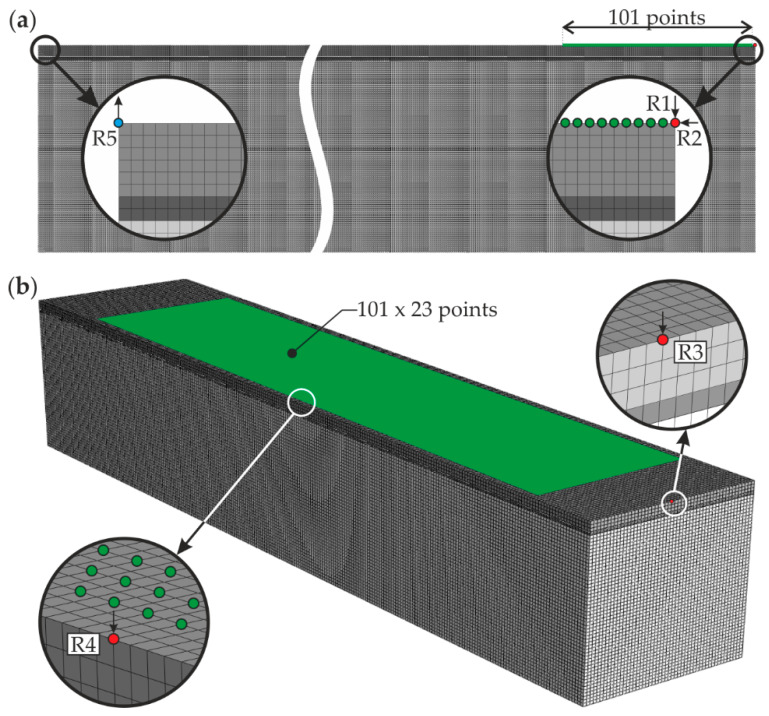
Numerical models for specimen #1: (**a**) 2-D plane model and (**b**) 3-D spatial model.

Two kinds of models were prepared for different purposes, plane (2-D) and spatial (3-D). Two-dimensional models (example in Figure 4a) were prepared for specimens #1 and #5 to analyze the differences between guided wave fields in a single-layer plate and a three-layer medium. Two-dimensional four-node plane strain elements with reduced integration (CPE4R) were used. The element size was constant and equal to 1 × 1 mm^2^ throughout the whole model. This value satisfies the requirement of the appropriate mapping of wave behavior (at least 10 nodes for the length of the shortest considered wave) presented in [48]. The calculations were conducted with a fixed time step with a value of 10^−7^ s that meets the recommendation of at least 20 integration points per cycle of the wave with the highest frequency of interest [49]. For initial analysis, the wave was excited at one end of the plate (point R1) and its signal was collected at another end (point R5) to determine the time-of-flight (TOF) of the wave through the length of the specimen. In the same simulation, guided wave field was saved for the whole model. The excitation had the form of a concentrated force varying in time in accordance with a 5-cycle tone burst with a center frequency of 10, 23, and 100 kHz (three different simulations). The plane models were also used to determine the numerical dispersion curves, similarly as in the experimental investigations (cf. Figure 3b). The input signal was applied in points R1 and R2 (independently) as a wave packet modulated from a single-cycle sine function with a different frequency (50, 100, 150, 200, and 250 kHz), resulting with ten consecutive simulations. The guided wave responses were obtained at 101 points distributed along the line with a length of 100 mm, giving 1 mm spacing between each point. It is worth noting that the measurement path was oriented perpendicularly to the one in experimental investigations. However, the results of both approaches could be compared, as long the materials used were modeled as isotropic and only the initial parts of signals were further analyzed. Three-dimensional models (example in Figure 4b) were prepared for specimens #1–#5 to verify the results of experimental measurements. Eight-node linear brick elements with reduced integration (C3D8R) and a global size of 2 mm were applied. The integration step was 2 × 10^−7^ s. Both element size and time step met the requirements from [48,49], described above. The scheme of excitation and collection of signals was identical with the experimental measurements (cf. Figure 3c). The wave (Hanning windowed five-cycle sine function with a carrier frequency of 100 kHz) was excited in point R3 or R4 and collected in 2323 points covering the area of 88 × 400 mm^2^.

### 2.4. Signal Processing with Weighted Root Mean Square

The direct analysis of the excited guided wave field usually does not give useful information about the shape, position and size of defects occurring in an examined structure. The utility of the results can be enhanced by further signal processing. The simple and widely used approach is based on the calculation of the effective value, also called the root mean square (RMS). For damage identification and imaging, it is advantageous to use the weighted variant of RMS (WRMS) that decreases the impact of the incident wave on the results obtained. The WRMS for a discrete time domain signal *s_r_* = *s*(*t_r_*) consisted of *N* values sampled with a constant Δ*t* interval can be calculated with respect to the formula [31,50,51,52,53]:

(1) $$WRMS=\sqrt{\frac{1}{N}\sum_{r=1}^{N}w_{r}s_{r}^{2}}$$

in which *w_r_* is a so-called weighting factor, defined as the function of the number of consecutive sample *r* as:

(2) $$w_{r}=r^{m},\;m\geq 0,\;r=1,2,\ldots ,N$$

where *m* is a non-negative power of the weighting factor. For *m* = 0 the above formulas describe the simple root mean square (without weighting). According to the literature cited above, the value of *m* was usually set arbitrary, however, it can be concluded that efficient results can be obtained for the higher values of *m*, e.g., *m* = 1 (linear weighting factor) or *m* = 2 (square variant). Further increasing of power *m* does not enhance the results in a significant manner.

### 2.5. Dispersion Curve Estimation with Matrix Pencil Method

The wavenumber–frequency relations can be calculated based on a set of wave propagation signals collected in a number of points spaced evenly along a measurement line. One of the popular methods is the use of the two-dimensional fast Fourier transform (2D-FFT [48,54,55]). However, the curves obtained are in the form of 2D maps which can be problematic when comparing different results. To solve this problem, a simple and robust algorithm called the Matrix Pencil (MP) technique [56,57,58] can be used. The method is based on the calculation of the one-dimensional fast Fourier transform (FFT). Its advantage is a good elimination of noise effects on the obtained results. Let us have the set of time-domain signals measured in the *m* points distributed along *z* axis with the interval Δ*z*. The Fourier coefficients *X*(*z*,*ω*) for each signal *x*(*z*,*t*) can be calculated with respect to the formula:

(3) $$X(z,\omega )=\int_{-\infty }^{\infty }x(z,t)e^{-i\omega t}dt$$

where *ω* is a circular frequency. Then, the wavenumbers are estimated using the forward/backward averaging technique. The sequence *x_n_* of *m* complex values at each fixed frequency *ω*_0_ is assumed to be the sum of *p* complex exponentials representing wavenumbers, as follows:

(4) $$x_{n}=x_{n}(\omega_{0})=X(z_{n},\omega_{0})=\sum_{j=1}^{p}a_{j}e^{-ik_{j}z},\;n=1,2,\ldots ,m.$$

In the above formula, *p* is the model order, so-called pencil parameter that should be chosen based on the unknown number of searched signal modes *q* and satisfy the limitation:

(5) q≤p≤m−q.

The algorithm requires the construction of a parent Hankel matrix **H** (*m* − *p* by *p* + 1) from the sequence *x_n_* as presented below:

(6) $$H=\left[\begin{array}{cccc} x_{1} & x_{2} & \cdots & x_{p+1}\\ x_{2} & x_{3} & \cdots & x_{p+2}\\ \vdots & \vdots & \ddots & \vdots \\ x_{m-p} & x_{m-p+1} & \cdots & x_{m} \end{array}\right].$$

Two submatrices of **H** need to be created, the **H**_0_ formed by deleting the first column of **H** and **H**_1_ formed by deleting the last column of **H**. The sets of forward *λ_f_* and backward *λ_b_* exponential estimates can be calculated by solving the two eigenvalue problems, respectively:

(7) $$\left(H_{0}^{+}H_{1}-\lambda_{f}I\right)e=0,\;\left(H_{1}^{+}H_{0}-\lambda_{b}I\right)e=0$$

where (.)^+^ indicates the Moore-Penrose generalized inverse and **I** is the identity matrix. The sets of forward *k_f_* and backward *k_b_* complex wavenumbers are computed as follows:

(8) $$k_{f}=\frac{\ln\lambda_{f}}{i\Delta z},\;k_{b}=-\frac{\ln\lambda_{b}}{i\Delta z}.$$

The final wavenumber *k* values can be calculated by the averaging technique presented in [56]. Firstly, the sets *k_f_* and *k_b_* are sorted and matched in pairs in the order of sorting. If the relative difference between two values of a certain pair is within a specified tolerance *l*, the pair is averaged arithmetically, if not, the pair is discarded.

## 3. Results and Discussion

### 3.1. Wave Propagation in Single-Layer and Multi-Layer Media

The first part of this section aimed at comparing the characteristics of guided waves propagating in a single layer plate (simulating the steel plate #5) and a two-layer plate (steel-adhesive) on a concrete half-space (three-layer medium modeling steel-adhesive-concrete composite beam #1). The materials for all the layers were assumed to be homogeneous, isotropic and linearly elastic (according to the material parameters presented in Table 1). Dispersion curves were calculated using a theoretical, numerical and experimental approach. The theoretical dispersion curves for the single-layer plate #5 were obtained in the way of solving well-known Rayleigh–Lamb equations in Matlab^®^. In the case of the three-layer medium, the theoretical curves were calculated using an open-source toolbox called Elastic-Matrix [59], based on the partial-wave method. Although the proposed algorithm is stated to be inaccurate for leaky cases, the appropriate solution can be obtained by selecting appropriate calculation parameters. The experimental and finite element method (FEM) numerical results were attained from the collected time-domain wave signals (91 and 101 signals, respectively) using the Matrix Pencil Method [56,57,58]. The pencil parameter and tolerance were chosen heuristically and equal to *p* = 16, *l* = 1.4% for the experimental analysis and *p* = 13, *l* = 2.2% for the numerical one. The determination of optimal values of these parameters requires further work; however, this aspect is not essential for the usefulness of results obtained in this research. The value of *p* was greater in the experimental calculation because the signal noise required computing more estimates, however, the tolerance needed to be more rigorous to exclude false results. The quality of results was enhanced by the authorial script deleting ‘lonely’ points. The complete wavenumber–frequency spectra were obtained by superimposing the dispersion curves calculated for the individual measurements with different location of the actuator and the excitation frequency.

**Figure 5:**
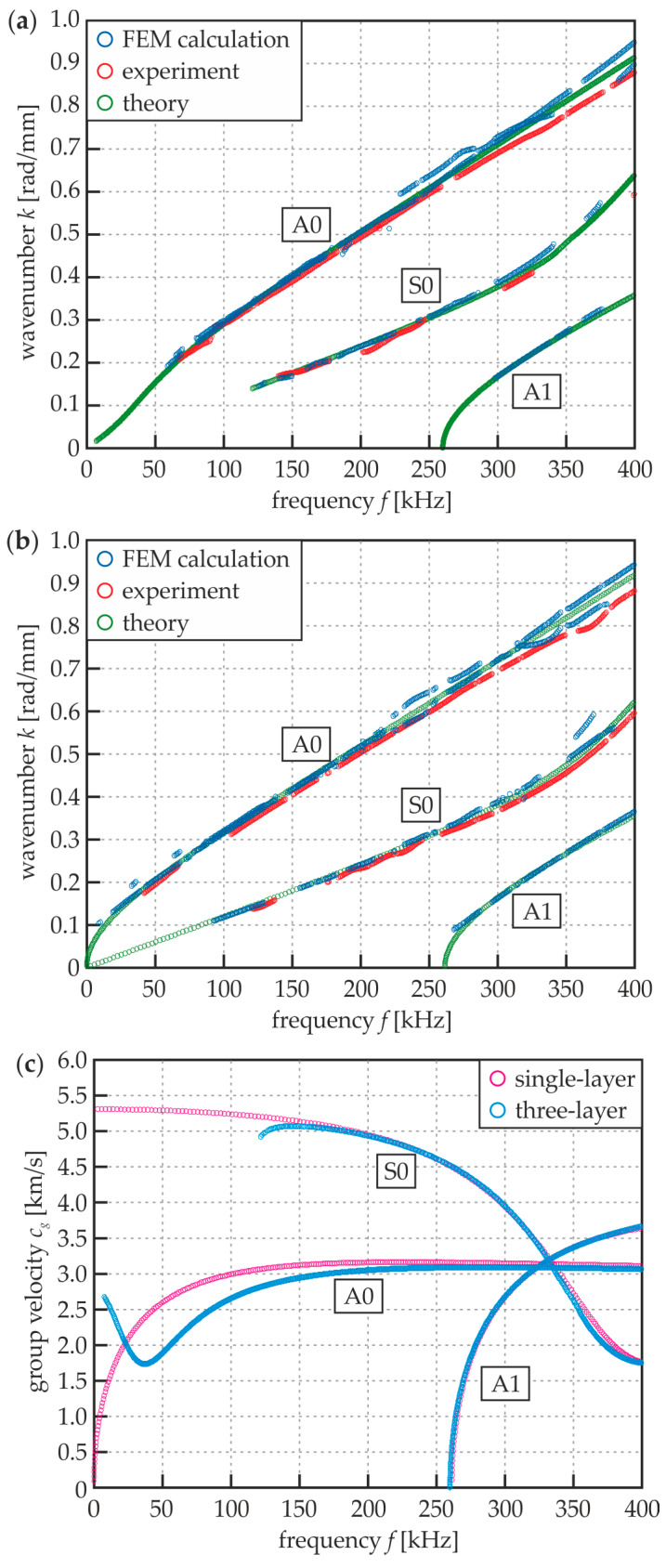
Dispersion curves for analyzed specimens: (**a**) wavenumber vs. frequency for three-layer plate #1; (**b**) wavenumber vs. frequency for single-layer steel plate #5; and (**c**) theoretical group velocity vs. frequency for three-layer plate #1 and single-layer plate #5.

The dispersion curves obtained from the three approaches are presented in Figure 5. The wavenumber–frequency relations for specimen #1 (Figure 5a) reveal the fact, that two flexural (A_0_, A_1_) and one longitudinal (S_0_) modes can propagate in the frequency range up to 400 kHz. It is essential to note, that the fundamental S_0_ mode does not appear in the range up to 122 kHz and the A_1_ mode is present for the frequencies above 260 kHz, thus only one mode can propagate for the assumed excitation frequency equal to 100 kHz. The comparison of the three approaches allowed concluding that the overall agreement between the numerical, experimental and theoretical results was very good. However, the higher mode A_1_ was not identified in the experimental approach, probably due to the setup limitations. It is also important to note that neither experimental nor numerical calculations detected A_0_ in the range up to 60 kHz, what could have been caused by the strict tolerance level. However, the increase of *l* would significantly decrease the legibility of the curves obtained. The analysis of dispersion curves for specimen #5 (Figure 5b) leads to similar conclusions. Compared with the results for beam #1, the S_0_ mode can propagate in the whole assumed frequency range, such as A_0_. The dispersion relations for both specimens for the frequencies above 150 kHz are comparable. The important differences are observable in the lower frequency range. To expose these dissimilarities, the theoretical dispersion curves for group velocity were prepared for both #1 and #5 specimens (Figure 5c). No significant difference can be seen for the A_1_ curve. The shapes of A_0_ and S_0_ curves are also very similar for the higher frequency range. As mentioned above, S_0_ mode is not present in the lower frequency range. To be noticed, it is important to analyze the shape of the A_0_ mode, because for the damage imaging, measurements the waves were excited perpendicularly to the plate surface and the out-of-plane velocity signals were collected. It is interesting that in the frequency range up to about 23 kHz it propagates with higher velocity in the specimen #1, whereas above this limit, the velocity of propagation is higher in specimen #5.

**Figure 6:**
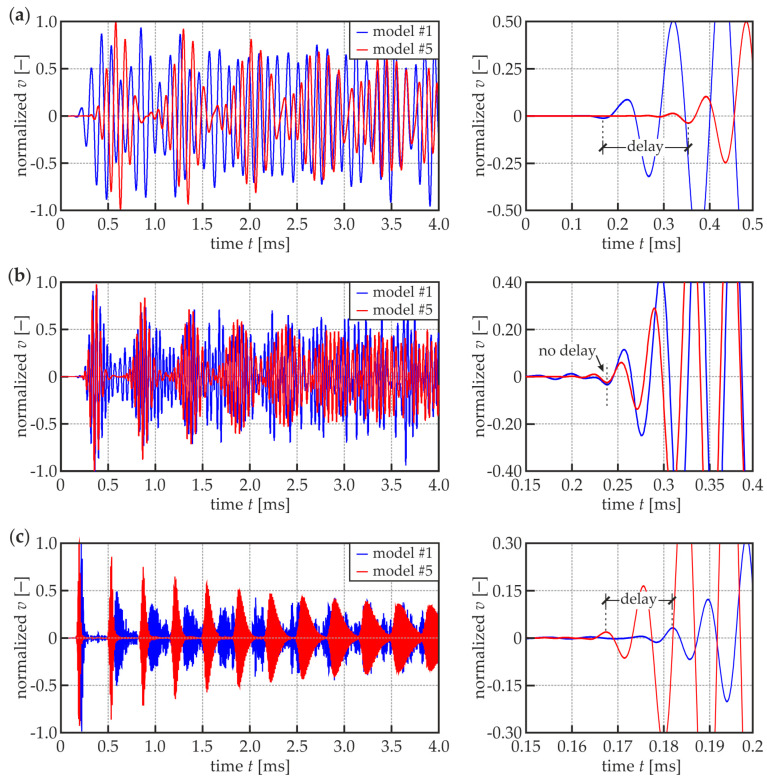
Normalized velocity signals (whole signals and zoom) registered at point R5 in numerical models of specimens #1 and #5 for different excitation frequencies: (**a**) 10 kHz; (**b**) 23 kHz; and (**c**) 100 kHz.

The simple FEM numerical simulations were performed to check whether this dispersion relationship is actually observable. Figure 6 shows the time signals of propagating waves in specimens #1 and #5 obtained from the numerical models in point R5 as the results of R1 excitation (cf. Figure 4a). It is clearly visible, (especially in close-ups) that the wave packet related to the A_0_ mode appears earlier in the signal from specimen #1 for the excitation frequency equal to 10 kHz (Figure 6a). If the excitation frequency is equal to 23 kHz, no difference in time of flight (TOF) of the first wave packet is observable (Figure 6b). The further increasing of the frequency value leads to the situation when the wave propagates faster in specimen #5 (Figure 6c). The TOF values for both models were calculated based on the dispersion curves (theoretical TOF) and are presented in Table 2, together with the values got from signals recorded. The relative differences between theoretical and numerical TOFs do not exceed a few percent, which indicates on the good compliance between dispersion curves and FEM simulations.

**Figure 7:**
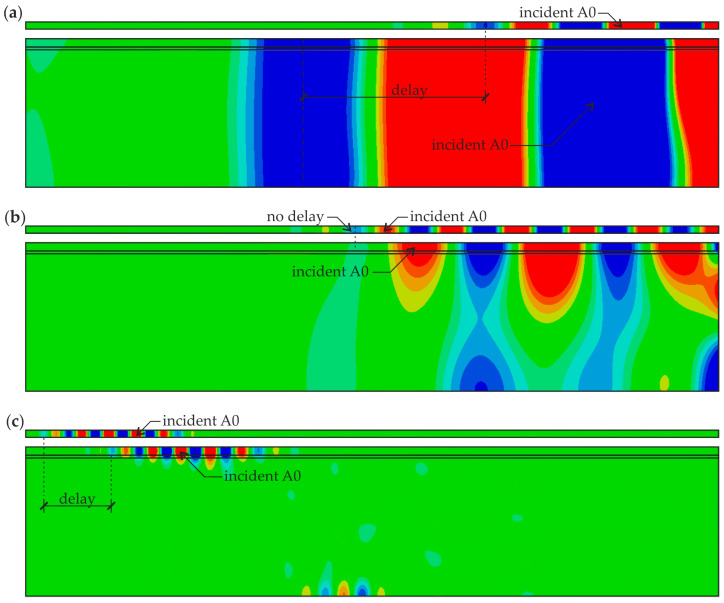
Numerical wave fields in 2-D finite element method (FEM) models of specimens #1 and #5 (*t* = 0.17 ms) for different excitation frequencies: (**a**) 10 kHz; (**b**) 23 kHz; and (**c**) 100 kHz.

In addition to the analysis of the time signals, the guided wave fields representing the propagation of A_0_ mode for specimens #1 and #5 for a specific time instance 0.17 ms are shown in Figure 7. It is visible that the incident wave with the frequency of 10 kHz propagates faster in beam #1 compared with plate #5 (Figure 7a), what stays in the agreement with the comparison of dispersion curves. What is also important, the wave propagates through the three-layer medium as a single waveguide, although the wave was excited only in the steel plate. It is possible because the A_0_ wavelength is large enough (271 mm), compared to the overall sample thickness (108 mm). The length of the A_0_ mode in beam #1 is significantly smaller (74 mm), but much greater than the thickness of the plate (6 mm). For the frequency equal to 23 kHz (Figure 7b) the wave packets in both specimens propagate with similar velocity. The distribution of the wave field trough the thickness of specimen #1 is not uniform, the amplitude decreases with the depth because the wavelength (107 mm) is slightly smaller than the specimen thickness. The interesting situation takes place when the excitation frequency increases to 100 kHz (Figure 7c). The wave propagation velocity in specimen #1 is smaller compared with specimen #5 (what is confirmed by the dispersion curves), but the wave propagates mainly in the steel plate and adhesive film. The reason is that the wavelength is now much smaller (22 mm) than the overall specimen thickness. For the concrete part, only the Rayleigh waves can be observed propagating at the interface between concrete and adhesive and the bottom boundary. The length of the wave propagating in plate #5 is comparable with the one present in the beam #1 and equal to 20 mm, which is also clearly visible in the snapshot. The analysis of the FEM simulations illustrating in Figure 7 allows concluding that the differences in the dispersion curves for both, three-layer and single-layer media are less pronounced for the higher frequency range (above about 120 kHz), because in this case, the wave propagates mainly in the steel plate, passing by the influence of the concrete part. Thus, the damage visualization in analyzed specimens would not be successful in this case. On the other hand, lower frequencies result in poorer image resolution. As the compromise, the excitation frequency equal to 100 kHz was chosen for further investigations aimed at damage detection and imaging.

### 3.2. WRMS-Based Damage Identification and Imaging

**Figure 8:**
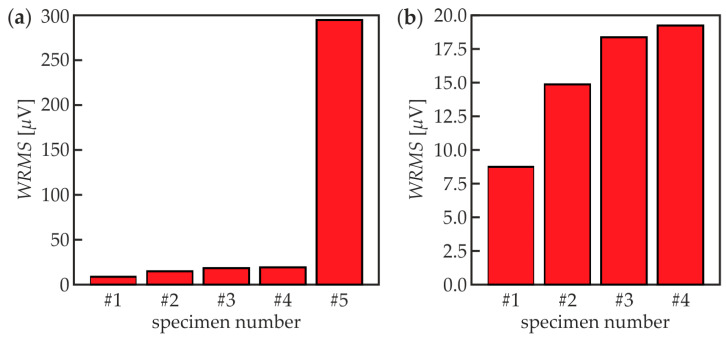
Weighted root mean square (WRMS) values of experimental signals collected in P5 point: (**a**) specimens #1–#5 and (**b**) specimens #1–#4.

The non-destructive diagnostics of specimens #1–#5 was conducted in two stages: identification and imaging. The aim of the damage identification was to detect, whether there are any defects in the adhesive joint between the concrete beam and the steel plate. The root mean squares values were calculated for the time signals excited by the actuator located in point P5 and measured by the sensor located in point P3 (see Figure 3c). This means that the wave propagated through the whole length of the specimen. The calculations were performed for the time of averaging assumed as *t_a_* = 1 ms, with a linear weighting factor (*m* = 1, *w_r_* = *r*). The results for all specimens are presented in Figure 8a. It is clearly visible that the WRMS value for specimen #5 (single-layer steel plate) is significantly higher than in others. In the case of specimen #5, the point of excitation was located on the free steel plate, characterized by the relatively small material damping. For specimens #1–#4, the wave was excited on the area where the steel plate had a good connection with the adhesive layer and concrete part (i.e., materials with higher damping) what resulted in the instantaneous leak and dissipation of energy in the bottom part of the specimen. For this reason, a close-up of the chart for the specimens #1–#4 is shown in Figure 8b. The correlation between WRMS value and the size of the defect is clearly visible, with the greater the damaged area, the higher WRMS value. This relationship occurs because the wave energy is magnified by the multiple reflections of propagating waves at the boundaries of defects. It is also worth noticing that the WRMS increased significantly between intact #1 and damaged #2 specimen (71%) but the further growth of defect surface did not result in the significant growth of WRMS. The damaged area of specimen #4 was 2.5 times bigger than in specimen #3 but the growths in comparison with #1 were equal to 116% and 129%, respectively. Concluding, the comparative analysis of WRMS values gave the answer about the presence of the defect, however, it did not provide the information about the size and location of damaged areas.

**Figure 9:**
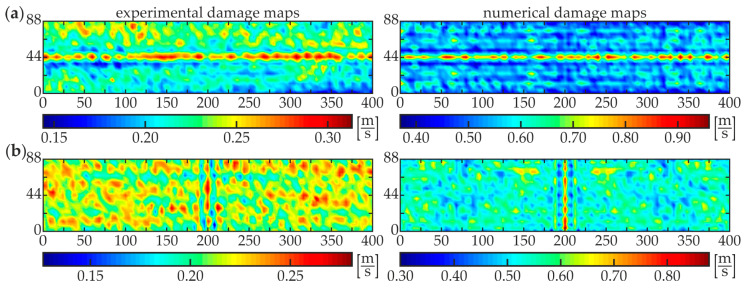
Experimental and numerical WRMS damage maps for specimen #1: (**a**) Excitation P3 and (**b**) Excitation P4.

**Figure 10:**
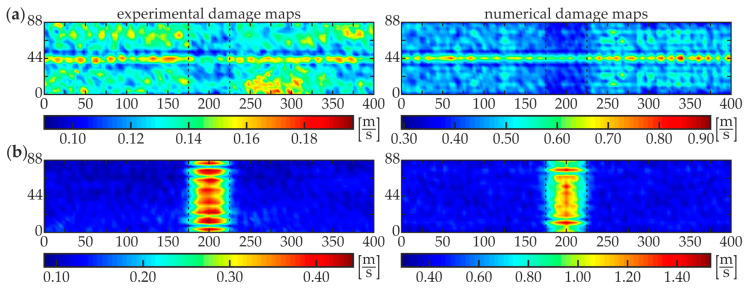
Experimental and numerical WRMS damage maps for specimen #2: (**a**) excitation P3 and (**b**) excitation P4.

The second stage, i.e., damage imaging, was performed to identify the exact position and size of the damaged area of each specimen. The WRMS map in the form of the two-dimensional defect representation over the scanned area was prepared based on the experimental investigations and numerical simulations on 3-D FEM models (see Figure 3c and Figure 4b). The parameters of the calculations were assumed to be the same as for damage identification stage (*t_a_* = 1 ms, *w_r_* = *r*). The results for specimens #1–#5 are presented below. Figure 9 shows the WRMS maps for the intact composite beam (specimen #1). Regardless of the location of the excitation point, the visualizations reveal that no internal defects were detected inside the analyzed adhesive joint. The values in each experimental and numerical map are concentrated over a certain value. The horizontal lines with magnified values in images for excitation P3 (Figure 9a), such as the vertical lines for excitation P4 (Figure 9b) are the effects of the specimen and the load symmetry. Some interesting conclusions can be derived from the analysis of the results for specimen #2 with a partial debonding (Figure 10). It is clearly visible that the map obtained for the excitation applied in point P3 (Figure 10a) results in the moderate defect imaging when compared with the excitation in point P4 (Figure 10b). The reason is that the wave excited in point P3 (in the area of good adhesion) strongly leaks into concrete, so the wave energy is damped significantly before meeting the boundaries of the defect, thus it has no possibility to be clearly visualized. It was already possible to conclude in the stage of damage identification (the high WRMS value for specimen #5 indicated on this effect, see Figure 8a). However, the damaged area is somehow detectable and it is characterized by slightly smaller WRMS values than the remaining part of the sample. The energy of the wave excited by the actuator P4 is concentrated in the damaged area and the damage map has a high quality. The properly boded areas have much lower WRMS values because the waves transmitted to the three-layer medium are highly damped.

**Figure 11:**
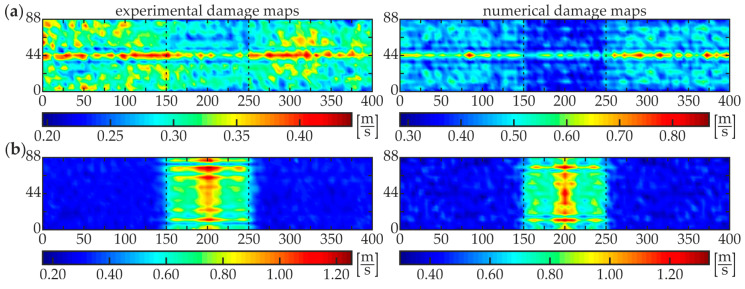
Experimental and numerical WRMS damage maps for specimen #3: (**a**) excitation P3 and (**b**) excitation P4.

**Figure 12:**
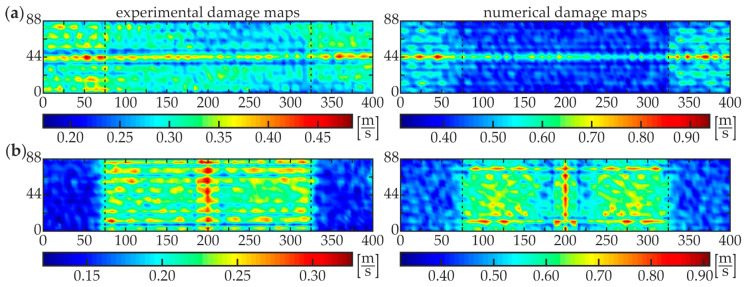
Experimental and numerical WRMS damage maps for specimen #4: (**a**) excitation P3 and (**b**) excitation P4.

**Figure 13:**
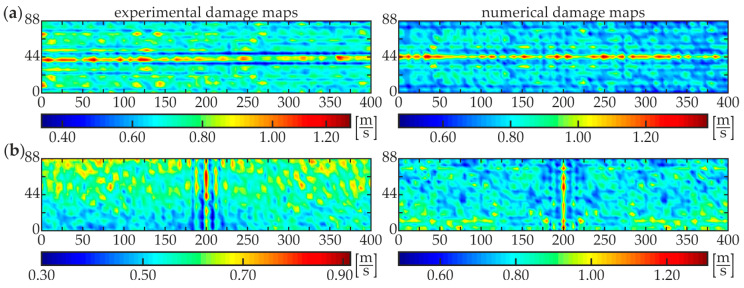
Experimental and numerical WRMS damage maps for specimen #5: (**a**) excitation P3 and (**b**) excitation P4.

A good agreement of experimental and numerical results is observed, however, the quality of the numerical visualization for the excitation in point R3 is richer compared with the experimental one. This may be caused by the noise present in the experimental signals. Similar conclusions can be derived from the analysis of damage maps of scanning for the two other partially damaged beams: #3 (Figure 11) and #4 (Figure 12). The WRMS maps for the fully debonded joint #5 (Figure 13) are similar to the ones obtained for the intact composite beam #1 (cf. Figure 9). This convergence allows stating that the damage visualization is possible only when the area of scanning covers both areas of good adhesion and debonding. The only difference between both specimens is that the value of WRMS is significantly higher for the steel plate (see color bars in Figure 9 and Figure 13).

## 4. Conclusions

This paper describes a WRMS-based ultrasonic non-destructive testing approach based on the scanning laser Doppler vibrometry technique. The damage identification and imaging in composite steel–concrete beams were performed. Based on the results of the experimental investigations evaluated by numerical FEM simulations and supported by theoretical analysis, the following final remarks can be formulated.

- The appropriate choice of the excitation frequency is an important issue for the effectiveness of proposed technique. The wave characteristics for a three-layer composite beam and a single-layer steel plate (simulating debonding) were similar for a frequency range above about 120 kHz, thus the damage imaging will not be effective in a higher frequency range. However, the lower frequencies were also not effective because of the decrease in image resolution.
- The comparable analysis of WRMS values calculated for the single wave signals allowed the initial detection of damaged specimens. However, the actual defect size, shape, and position were indeterminable at this stage.
- The main factor affecting the efficiency of damage imaging was the phenomenon of wave leakage and dissipation of the energy, therefore, the position of the excitation point was crucial in the context of debonding detection. The scanning with the actuator placed directly on the damaged area (single-layer steel plate), where the wave leakage did not occur, gave more valuable results. However, the results obtained for the excitation in the area of good adhesion (three-layer composite beam) were also useful, despite the more intensive wave leakage into the concrete beam.
- The visualization of defects was possible only when both debonded and properly connected areas were covered by the scanning region. If the image did not have areas with significantly different values, it was not clear whether the entire analyzed area was well-bonded or debonded.

The leading conclusion of the presented research is that the guided wave propagation method supported with WRMS calculation can be successfully applied for damaged imaging in steel–concrete composite beams. However, considering the complexity of guided wave propagation phenomenon, it is important to perform some initial analysis, including dispersion curves determination and FEM numerical calculations. Further works will be directed to develop the proposed method to be applicable for non-destructive diagnostics of real-scale engineering structures.

## Figures and Tables

**Table 1:** Material parameters for each material in numerical simulations.

Material	Density *ρ* (kg/m^3^)	Elastic Modulus *E* (GPa)	Poisson’s Ratio *ν* (–)
concrete	2364.4	49.5	0.12
steel	7822.8	200.3	0.30
adhesive	1611.8	14.9	0.30

**Table 2:** Time of flight of the wave packet based on the dispersion curves and the wave signals.

Frequency *f* (kHz)	Theoretical TOF (#1) (*μ*s)	Numerical TOF (#1) (*μ*s)	Theoretical TOF (#5) (*μ*s)	Numerical TOF (#5) (*μ*s)
10	193	177	344	358
23	246	258	247	255
100	189	183	167	168

## References

[1] Zhao X.L., Zhang L. (2007). State-of-the-art review on FRP strengthened steel structures. Eng. Struct..

[2] De Lorenzis L., Teng J.G. (2007). Near-surface mounted FRP reinforcement: An emerging technique for strengthening structures. Compos. Part B Eng..

[3] Teng J.G., Yu T., Fernando D. (2012). Strengthening of steel structures with fiber-reinforced polymer composites. J. Constr. Steel Res..

[4] Czaderski C., Meier U. (2018). EBR strengthening technique for concrete, long-term behaviour and historical survey. Polymers.

[5] Barnes R.A., Mays G.C. (2001). The transfer of stress through a steel to concrete adhesive bond. Int. J. Adhes. Adhes..

[6] Ali M.S.M., Oehlers D.J., Bradford M.A. (2005). Debonding of steel plates adhesively bonded to the compression faces of RC beams. Constr. Build. Mater..

[7] Bez Batti M.M., do Vale Silva B., Piccinini Â.C., dos Santos Godinho D., Antunes E.G.P. (2018). Experimental analysis of the strengthening of reinforced concrete beams in shear using steel plates. Infrastructures.

[8] Alam M.A., Onik S.A., Mustapha K.N. (2020). Crack based bond strength model of externally bonded steel plate and CFRP laminate to predict debonding failure of shear strengthened RC beams. J. Build. Eng..

[9] Giurgiutiu V., Lyons J., Petrou M., Laub D., Whitley S. (2001). Fracture mechanics testing of the bond between composite overlays and a concrete substrate. J. Adhes. Sci. Technol..

[10] Schilde K., Seim W. (2007). Experimental and numerical investigations of bond between CFRP and concrete. Constr. Build. Mater..

[11] Pan J., Leung C.K.Y., Luo M. (2010). Effect of multiple secondary cracks on FRP debonding from the substrate of reinforced concrete beams. Constr. Build. Mater..

[12] Napoli A., Realfonzo R. (2015). Reinforced concrete beams strengthened with SRP/SRG systems: Experimental investigation. Constr. Build. Mater..

[13] Gao P., Gu X., Mosallam A.S. (2016). Flexural behavior of preloaded reinforced concrete beams strengthened by prestressed CFRP laminates. Compos. Struct..

[14] Mertoğlu Ç., Anil Ö., Durucan C. (2016). Bond slip behavior of anchored CFRP strips on concrete surfaces. Constr. Build. Mater..

[15] Akroush N., Almahallawi T., Seif M., Sayed-Ahmed E.Y. (2017). CFRP shear strengthening of reinforced concrete beams in zones of combined shear and normal stresses. Compos. Struct..

[16] Ascione F., Lamberti M., Napoli A., Realfonzo R. (2020). Experimental bond behavior of Steel Reinforced Grout systems for strengthening concrete elements. Constr. Build. Mater..

[17] Zhang P., Lei D., Ren Q., He J., Shen H., Yang Z. (2020). Experimental and numerical investigation of debonding process of the FRP plate-concrete interface. Constr. Build. Mater..

[18] Lai W.L., Lee K.K., Kou S.C., Poon C.S., Tsang W.F. (2012). A study of full-field debond behaviour and durability of CFRP-concrete composite beams by pulsed infrared thermography (IRT). NDT E Int..

[19] Tashan J., Al-Mahaidi R. (2014). Bond defect detection using PTT IRT in concrete structures strengthened with different CFRP systems. Compos. Struct..

[20] Yi Q., Tian G.Y., Yilmaz B., Malekmohammadi H., Laureti S., Ricci M., Jasiuniene E. (2019). Evaluation of debonding in CFRP-epoxy adhesive single-lap joints using eddy current pulse-compression thermography. Compos. Part B Eng..

[21] Yazdani N., Beneberu E., Riad M. (2019). Nondestructive Evaluation of FRP-Concrete Interface Bond due to Surface Defects. Adv. Civ. Eng..

[22] Gu J.C., Unjoh S., Naito H. (2020). Detectability of delamination regions using infrared thermography in concrete members strengthened by CFRP jacketing. Compos. Struct..

[23] Shiotani T., Momoki S., Chai H., Aggelis D.G. (2009). Elastic wave validation of large concrete structures repaired by means of cement grouting. Constr. Build. Mater..

[24] Rucka M., Wilde K. (2015). Ultrasound monitoring for evaluation of damage in reinforced concrete. Bull. Polish Acad. Sci. Tech. Sci..

[25] Choi H., Ham Y., Popovics J.S. (2016). Integrated visualization for reinforced concrete using ultrasonic tomography and image-based 3-D reconstruction. Constr. Build. Mater..

[26] Zielińska M., Rucka M. (2018). Non-Destructive Assessment of Masonry Pillars using Ultrasonic Tomography. Materials.

[27] Słoński M., Schabowicz K., Krawczyk E. (2020). Detection of Flaws in Concrete Using Ultrasonic Tomography and Convolutional Neural Networks. Materials.

[28] Garbacz A., Piotrowski T., Courard L., Kwaśniewski L. (2017). On the evaluation of interface quality in concrete repair system by means of impact-echo signal analysis. Constr. Build. Mater..

[29] Sadowski Ł., Hoła J., Czarnecki S. (2016). Non-destructive neural identification of the bond between concrete layers in existing elements. Constr. Build. Mater..

[30] Marks R., Clarke A., Featherston C., Paget C., Pullin R. (2016). Lamb Wave Interaction with Adhesively Bonded Stiffeners and Disbonds Using 3D Vibrometry. Appl. Sci..

[31] Rucka M., Wojtczak E., Lachowicz J. (2018). Damage Imaging in Lamb Wave-Based Inspection of Adhesive Joints. Appl. Sci..

[32] Wojtczak E., Rucka M. (2019). Wave frequency effects on damage imaging in adhesive joints using lamb waves and RMS. Materials.

[33] Castaings M., Hosten B., François D. (2004). The sensitivity of surface guided modes to the bond quality between a concrete block and a composite plate. Ultrasonics.

[34] Shen Y., Hirose S., Yamaguchi Y. (2014). Dispersion of ultrasonic surface waves in a steel-epoxy-concrete bonding layered medium based on analytical, experimental, and numerical study. Case Stud. Nondestruct. Test. Eval..

[35] Zeng L., Parvasi S.M., Kong Q., Huo L., Lim I., Li M., Song G. (2015). Bond slip detection of concrete-encased composite structure using shear wave based active sensing approach. Smart Mater. Struct..

[36] Song H., Popovics J.S. (2017). Characterization of steel–concrete interface bonding conditions using attenuation characteristics of guided waves. Cem. Concr. Compos..

[37] Li J., Lu Y., Guan R., Qu W. (2017). Guided waves for debonding identification in CFRP-reinforced concrete beams. Constr. Build. Mater..

[38] Zima B., Rucka M. (2017). Guided wave propagation for assessment of adhesive bonding between steel and concrete. Procedia Eng..

[39] Rucka M. (2018). Failure Monitoring and Condition Assessment of Steel–concrete Adhesive Connection Using Ultrasonic Waves. Appl. Sci..

[40] Chen H., Xu B., Wang J., Luan L., Zhou T., Nie X., Mo Y.L. (2019). Interfacial debonding detection for rectangular cfst using the masw method and its physical mechanism analysis at the meso-level. Sensors.

[41] Liu S., Sun W., Jing H., Dong Z. (2019). Debonding Detection and Monitoring for CFRP Reinforced Concrete Beams Using Pizeoceramic Sensors. Materials.

[42] Ke Y.T., Cheng C.C., Lin Y.C., Huang C.L., Hsu K.T. (2019). Quantitative assessment of bonding between steel plate and reinforced concrete structure using dispersive characteristics of lamb waves. NDT E Int..

[43] Yan J., Zhou W., Zhang X., Lin Y. (2019). Interface monitoring of steel–concrete–steel sandwich structures using piezoelectric transducers. Nucl. Eng. Technol..

[44] Giri P., Mishra S., Clark S.M., Samali B. (2019). Detection of gaps in concrete–metal composite structures based on the feature extraction method using piezoelectric transducers. Sensors.

[45] Ng C.T., Mohseni H., Lam H.F. (2019). Debonding detection in CFRP-retrofitted reinforced concrete structures using nonlinear Rayleigh wave. Mech. Syst. Signal. Process..

[46] Wang Y., Li X., Li J., Wang Q., Xu B., Deng J. (2019). Debonding damage detection of the CFRP-concrete interface based on piezoelectric ceramics by the wave-based method. Constr. Build. Mater..

[47] Huo L., Cheng H., Kong Q., Chen X. (2019). Bond-slip monitoring of concrete structures using smart sensors—A review. Sensors.

[48] Alleyne D., Cawley P. (1991). A two-dimensional Fourier transform method for the measurement of propagating multimode signals. J. Acoust. Soc. Am..

[49] Moser F., Jacobs L.J., Qu J. (1999). Modeling elastic wave propagation in waveguides with the finite element method. NDT E Int..

[50] Żak A., Radzieński M., Krawczuk M., Ostachowicz W. (2012). Damage detection strategies based on propagation of guided elastic waves. Smart Mater. Struct..

[51] Lee C., Zhang A., Yu B., Park S. (2017). Comparison study between RMS and edge detection image processing algorithms for a pulsed laser UWPI (Ultrasonic wave propagation imaging)-based NDT technique. Sensors.

[52] Pieczonka Ł., Ambroziński Ł., Staszewski W.J., Barnoncel D., Pérès P. (2017). Damage detection in composite panels based on mode-converted Lamb waves sensed using 3D laser scanning vibrometer. Opt. Lasers Eng..

[53] Kudela P., Wandowski T., Malinowski P., Ostachowicz W. (2016). Application of scanning laser Doppler vibrometry for delamination detection in composite structures. Opt. Lasers Eng..

[54] Harb M.S., Yuan F.G. (2015). A rapid, fully non-contact, hybrid system for generating Lamb wave dispersion curves. Ultrasonics.

[55] Gauthier C., Galy J., Ech-Cherif El-Kettani M., Leduc D., Izbicki J.L. (2018). Evaluation of epoxy crosslinking using ultrasonic Lamb waves. Int. J. Adhes. Adhes..

[56] Ekstrom M.P. (1996). Dispersion Estimation from Borehole Acoustic Arrays Using a Modified Matrix Pencil Algorithm. IEEE Proc. ASILOMAR-29.

[57] Mazzotti M., Bartoli I., Castellazzi G., Marzani A. (2014). Computation of leaky guided waves dispersion spectrum using vibroacoustic analyses and the Matrix Pencil Method: A validation study for immersed rectangular waveguides. Ultrasonics.

[58] Chang C.Y., Yuan F.G. (2018). Extraction of guided wave dispersion curve in isotropic and anisotropic materials by Matrix Pencil method. Ultrasonics.

[59] Ramasawmy D.R., Cox B.T., Treeby B.E. (2020). ElasticMatrix: A MATLAB toolbox for anisotropic elastic wave propagation in layered media. SoftwareX.

